# Clinical characteristics associated with elevated levels of lipoprotein(a) in patients with vascular risk

**DOI:** 10.1515/almed-2023-0150

**Published:** 2023-12-01

**Authors:** Javier Rubio-Serrano, Alejandra Gullón Ojesto, Carmen Suárez Fernández

**Affiliations:** La Princesa Biomedical Research Foundation, La Princesa Research Institute, Madrid, Spain; Unit of Vascular Risk, Service of Internal Medicine, La Princesa University Hospital, Madrid, Spain

**Keywords:** cardiovascular disease, risk factors, familial hypercholesterolemia, lipoprotein(a)

## Abstract

**Objectives:**

Lipoprotein(a) (Lp(a)) is increasingly used in the evaluation of patients with vascular risk due to its association with cardiovascular events. The purpose of this study was to identify the clinical characteristics of patients with elevated levels of Lp(a) attended in an outpatient vascular risk unit.

**Methods:**

An observational, retrospective study was conducted to assess the clinical characteristics of patients with elevated levels of Lp(a) (≥50 mg/dL), as compared to patients with normal values (<50 mg/dL). The sample was composed of 878 patients identified as having a high vascular risk due to a diagnosis of vascular disease, attended in a vascular risk unit between 2021 and 2022.

**Results:**

The highest levels of Lp(a) were independently associated with a higher probability of having a history of peripheral arterial disease (p=0.024), polygenic familial hypercholesterolemia (PH, p=0.030) and combined familial hypercholesterolemia (CFH, p=0.015); and using PCSK9 inhibitor treatment (p=0.029) and combination therapy with statins and ezetimibe (p=0.018). In contrast, there were no significant differences in relation to familial history of early cardiovascular disease (p=0.143) or personal history of cardiovascular disease (p=0.063), which contrasts with other series.

**Conclusions:**

Elevated levels of Lp(a) were associated with a history of peripheral arterial disease, diagnosis of FHP and CFH, and need for more intense lipid-lowering treatments.

## Introduction

Lipoprotein(a) (Lp(a)) is a spherical macromolecular complex consisting of a low-density lipoprotein-like particle. Lp(a) is composed of one molecule of apolipoprotein B100 (apoB100) bound covalently to apolipoprotein(a) (apo(a)) by a disulfide bridge [1, 2].

There is broad variability in plasma concentrations of Lp(a) in the general population. However, lifetime within-subject variability is limited [3]. Elevated levels of Lp(a) are associated with a higher risk for atherosclerotic cardiovascular disease and aortic stenosis. The risk increases in the presence of other risk factors, including hypertension, diabetes, obesity, smoking, or a family history of early cardiovascular disease [1, 4, 5].

Concentrations >30 mg/dL and >50 mg/dL contributed to increased cardiovascular risk mediated by different mechanisms [6]. Firstly, Lp(a) promotes the development of atheromatous plaques due to the binding of apo(a) to plasmin [7]. Additionally, Lp(a) is involved in the activation of proinflammatory signaling pathways and the reduction of endothelial nitric oxide availability [8, 9]. Lp(a) also interferes with fibrinolysis by binding to plasmin, thereby reducing its activity to dissolve clots on the inner surface of blood vessels. This interference may increase the risk for thrombi and arterial obstruction, thereby contributing to the development of cardiovascular diseases [10].

Previous diseases have demonstrated a higher prevalence of elevated levels of Lp(a) in patients with familial hypercholesterolemia (FH). FH is a hereditary genetic disorder characterized by elevated levels of LDL cholesterol (LDL-C) that increases the risk for atherosclerotic cardiovascular disease [11, 12]. This metabolic disorder is mainly caused by pathogenic mutations in the genes encoding the LDL receptor (LDLR) [13, 14]; proprotein convertase subtilisin/kexin type 9 (PCSK9) [14, 15]; or apoliprotein B (APOB) [14, 16].

There are not pharmacological treatments currently available for the reduction of Lp(a). However, measurement of Lp(a) concentrations in vascular risk patients may contribute to stratifying risk and modifying therapeutic targets [17]. The Spanish Association of Arteriosclerosis (SEA) recommends measuring Lp(a) at least once in life in patients with vascular risk, due to its role as a potent prognostic marker [18]. The purpose of this study was to identify the clinical characteristics of patients with elevated levels of Lp(a) attended in an outpatient vascular risk unit.

## Materials and methods

### Type of study

An observational, retrospective study was carried out involving patients with vascular risk attending the outpatient consultations of La Princesa University Hospital, a tertiary hospital in Madrid, Spain, serving a population of 323,000. Patients were included for two consecutive years, 2021 and 2022.

### Selection of patients

Sequential sampling was performed of all vascular-risk patients with a Lp(a) measurement available (with or without a history of cardiovascular events) attended in the outpatient consultations of internal medicine, cardiology, endocrinology, and neurology between 2021 and 2022. A total of 878 patients met the inclusion criteria.

This study was approved by the Ethics Committee of La Princesa University Hospital. The personal data of patients were always kept confidential.

### Study variables

The clinical and laboratory variables collected during routine visits included: demographics (age and sex); Lp(a) test requesting service; vascular risk factors (history of dyslipidemia, hypertension, diabetes, smoking, obesity, and family history of early cardiovascular disease); history of previous cardiovascular disease (ischemic heart disease, cerebrovascular disease, aortic stenosis, and peripheral arterial disease); diagnosis of familial dyslipidemia (differentiating between heterozygous familial hypercholesterolemia (HFH), polygenic familial hypercholesterolemia (PFH), and combined familial hypercholesterolemia (CHF)); Lp(a) (mg/dL) and LDL-C (mg/dL) levels in blood; and finally lipid-lowering treatments used: high-potency statins (atorvastatin ≥40 mg or rosuvastatin ≥10 mg), low-potency statins (atorvastatin <40 mg, rosuvastatin <10 mg, simvastatin, pravastatin, lovastatin, fluvastatin, or pitavastatin), ezetimibe, and/or PCSK9 inhibitors (PCSK9i).

The laboratory techniques currently used for determination of Lp(a) may be subject to variability due to heterogeneity in the size of the apo(a) molecule, determined by the number of type 2 kringle 4 domains in its encoding gene. Lp(a) quantification was carried out by immunoturbidimetry on a Roche/Hitachi Cobas c 701/702 analyzer with anti-apoA antibodies, as they are standardized quantification techniques against an apo(a) size-independent method. Levels were expressed in nmol/L, which were converted into mg/dL for clinical interpretation purposes.

### Statistical analysis

According to the literature, optimal plasma Lp(a) concentrations are <30 mg/dL. However, in clinical practice, Lp(a) levels >50 mg/dL have been observed to be strongly associated with a higher risk for cardiovascular disease [19], [20], [21]. A comparative analysis was performed of the clinical characteristics of patients with elevated levels of Lp(a) (Lp(a)≥50 mg/dL) and patients with levels associated with a lower risk for cardiovascular disease (Lp(a)<50 mg/dL).

Quantitative variables are presented as central tendency measures, means and standard deviations, whereas qualitative variables are expressed as frequencies and absolute percentages.

On univariate analysis, as data distribution was nonparametric, Mann–Whitney U test was performed to explore differences between categorical and continuous variables. Chi-square test was performed to assess differences in categorical variables (chi-square). Independent variables with a p-value <0.1 on univariate analysis were included for multivariate analysis. Clinically relevant variables associated with Lp(a) levels in the literature were also included for multivariate analysis [4]. For multivariate modeling, backward logistic regression was performed. An alpha error <0.05 with a 95 % confidence interval was considered statistically significant. All statistical analyses were performed using R version 4.3.0 software package [22].

## Results

A total of 878 patients with high cardiovascular risk attended between 2021 and 2022 were included in the study. Lp(a) testing was requested by the units of internal medicine (48 %), cardiology (46 %), endocrinology (2 %), neurology (1 %), and other services (3 %). In total, 274 patients (27.2 %) exhibited elevated levels of Lp(a) (Lp(a)≥50 mg/dL). Among patients with elevated Lp(a), 17 (1.9 %) had Lp(a)≥180 mg/dL, which has been associated with a lifetime risk for vascular events similar to that associated with HFH [23]. In addition, in 84 patients (10.7 %), levels ranged from 30 to 50 mg/dL and the remaining 520 patients (62.1 %) had levels <30 mg/dL, which are considered normal levels in the general population [24].

Mean age was 61 years, and 37 % were women. In relation to vascular risk factors, more than half of the participants had dyslipidemia and hypertension. Most patients had a high body mass index (BMI) consistent with overweight (27.3 %), and only 28.4 % had a family history of early cardiovascular disease. More than half of the sample had a history of vascular event, primarily ischemic heart disease (48.2 %), followed by cerebrovascular disease (7.4 %), peripheral arterial disease (6.3 %) and aortic stenosis (2.3 %). In total, 15.9 % of patients had a family history of dyslipidemia, most frequently, polygenic familial hypercholesterolemia. Finally, considering the lipid-lowering treatments used, more than half of the sample took high-potency statins (53.4 %); statins + ezetimibe (33.1 %); statins + iPCSK9 (2.8 %); and statins + ezetimibe + iPCSK9 (1.7 %) (Table 1).

**Table 1:** Clinical and antropometric variables of the study population.

Variables	Total population (n=878)	Lp(a)<50 mg/dL (n=604)	Lp(a)≥50 mg/dL (n=274)
Age, years, mean ± SD	60.8 ± 14.0	60.4 ± 13.9	61.8 ± 14.2
Sex, female, n (%)	321 (36.6)	209 (34.6)	112 (40.9)
Vascular risk factors			
Dyslipemia, n (%)	674 (76.8)	449 (74.3)	225 (82.1)
LDL-C, mg/dL, mean ± SD	90.2 ± 40.1	90.7 ± 40.8	89.1 ± 38.5
Hypertension, n (%)	516 (58.8)	361 (59.8)	155 (56.6)
Diabetes, n (%)	202 (23.0)	136 (22.5)	66 (24.1)
Active smoking, n (%)	212 (24.1)	160 (26.7)	52 (19.1)
BMI, mean ± SD	27.3 ± 4.7	27.5 ± 4.8	26.9 ± 4.6
FH of ECVD, n (%)^a^	129 (28.4)	82 (26.6)	47 (32.0)
Previous cardiovascular disease			
Total CVD, n (%)	479 (54.6)	329 (54.5)	150 (54.7)
Ischemic heart disease, n (%)	423 (48.2)	291 (48.2)	132 (48.2)
Cerebrovascular disease, n (%)	65 (7.4)	48 (8.0)	17 (6.2)
Aortic stenosis, n (%)	20 (2.3)	14 (2.3)	6 (2.2)
PAD, n (%)	55 (6.3)	30 (5.0)	25 (9.1)
Familial dyslipidemia			
HFH, n (%)	28 (3.2)	15 (2.5)	13 (4.7)
PFH, n (%)	93 (10.6)	50 (8.3)	43 (15.7)
FCH, n (%)	18 (2.1)	8 (1.3)	10 (3.7)
Lipid-lowering treatments			
High-potency statins, n (%)	469 (53.4)	310 (51.3)	159 (58.0)
Low-potency statins, n (%)	179 (20.4)	116 (19.2)	63 (23.0)
Ezetimibe, n (%)	326 (37.1)	195 (32.3)	131 (47.8)
PCSK9i, n (%)	47 (5.4)	20 (3.3)	27 (9.9)
Statins + ezetimibe, n (%)	291 (33.1)	172 (28.5)	119 (43.4)
Statins + PCSK9i, n (%)	25 (2.8)	10 (1.7)	15 (5.5)
Statins + ezetimibe + PCSK9i, n (%)	15 (1.7)	4 (0.7)	11 (4.0)

SD, standard deviation; LDL-C, low-density lipoprotein cholesterol; BMI, body mass index; FH of ECVD, family history of early cardiovascular disease; CVD, cardiovascular disease; PAD, peripheral arterial disease; HFH, heterozygous familial hypercholesterolemia; PFH, polygenic familial hypercholesterolemia; CFH, combined familial hypercholesterolemia, PCSK9i, protein convertase subtilisin kexin type 9 inhibitors. ^a^423 missing data; sample size was 455 patients, of which 147 had elevated levels of Lp(a) and 308 had normal levels.

Univariate analysis revealed an association between elevated levels of Lp(a) and dyslipidemia and a lower probability of being an active smoker. With regard to other risk factors, including familial history of early cardiovascular disease, no statistically significant differences were observed. In relation to history of vascular diseases, elevated levels of Lp(a) were associated with a history of peripheral arterial disease. In contrast, no association was found with other cardiovascular events (Tables 1 and 2).

**Table 2:** Univariate and multivariate analysis.

Variables	Univariate analysis		Multivariate analysis	
	Odds ratio	p-Value	Adjusted odds ratio	p-Value
Age, years	–	0.167	–	–
Sex (female)	0.8 (0.6–1.0)	0.087	–	–
Vascular risk factors				
Dyslipemia	1.6 (1.1–2.3)	0.015^a^	1.2 (0.7–2.3)	0.486
LDL-C, mg/dL	–	0.674	–	–
Hypertension	0.9 (0.7–1.2)	0.413	–	–
Diabetes	1.1 (0.8–1.5)	0.670	–	–
Active smoking	0.7 (0.5–0.9)	0.019^a^	0.6 (0.3–1.0)	0.080
BMI	–	0.100	–	–
FH of ECVD	1.3 (0.8–2.0)	0.283	1.4 (0.9–2.3)	0.143
Previous cardiovascular disease				
Total CVD	1.0 (0.8–1.4)	0.998	0.3 (0.1–1.0)	0.063
Ischemic heart disease	1.0 (0.8–1.3)	1.000	2.4 (0.7–9.0)	0.167
Cerebrovascular disease	0.8 (0.2–1.3)	0.439	–	–
Aortic stenosis	1.0 (0.3–2.4)	1.000	–	0.986
PAD	1.9 (1.1–3.3)	0.027^a^	3.4 (1.2–10.4)	0.024^a^
Familial dyslipidemia				
HFH	2.0 (0.9–4.2)	0.119	0.9 (0.3–2.5)	0.814
PFH	2.1 (1.3–3.2)	0.001^b^	1.9 (1.1–3.5)	0.030^a^
CFH	2.8 (1.1–7.5)	0.046^a^	3.7 (1.3–11.1)	0.015^a^
Lipid-lowering treatments				
High-potency statins	1.3 (1.0–1.8)	0.076	–	–
Low-potency statins	1.3 (0.9–1.8)	0.230	–	–
Ezetimibe	1.9 (1.4–2.6)	<0.001^c^	0.6 (0.2–1.7)	0.360
PCSK9i	3.2 (1.8–5.9)	<0.001^c^	5.0 (1.2–22.9)	0.029^a^
Statins + ezetimibe	1.9 (1.4–2.6)	<0.001^c^	4.4 (1.4–1.6)	0.018^a^
Statins + PCSK9i	3.4 (1.5–8.0)	0.003^b^	1.2 (0.1–1.3)	0.884
Statins + ezetimibe + PCSK^a^9i	6.1 (2.0–22.9)	0.001^b^	0.5 (0.0–5.8)	0.580

LDL-C, low-density lipoprotein cholesterol; BMI, body mass index; FH of ECVD, family history of early cardiovascular disease; CVD, cardiovascular disease; PAD, peripheral arterial disease; HFH, heterozygous familial hypercholesterolemia; PFH, polygenic familial hypercholesterolemia; CFH, combined familial hypercholesterolemia, PCSK9i, protein convertase subtilisin kexin type 9 inhibitors. ^a^p<0.05. ^b^p<0.01. ^c^p<0.001.

The percentage of patients with PFH and CHF was higher in the group of patients with elevated levels of Lp(a) (15.7 and 3.7 % respectively). However, the prevalence of HFH was similar in the two study populations.

Concerning the lipid-lowering treatments administered, the use of ezetimibe and iPCSK9 was significantly more frequent in the subgroup of patients with elevated levels of Lp(a): 47.8 vs. 32.3 % for ezetimibe, and 9.9 vs. 3.3 % for iPCSK9. Likewise, the combination of lipid-lowering drugs was significantly more frequent in the group of patients with elevated levels of Lp(a), being 43.4 vs. 28.5 % for the use of statins + ezetimibe; 5.5 vs. 1.7 % for statins + iPCSK9; and 4.0 vs. 0.7 % for statins + ezetimibe + iPCSK9 (Tables 1 and 2).

Finally, the variables with a p-value >0.05 on univariate analysis and considered to be clinically relevant in the literature (familial history of early cardiovascular disease, previous cardiovascular disease, ischemic heart disease, aortic stenosis, and HFH) were included in a multivariate logistic regression model. Analyses confirmed an independent relation of elevated levels of Lp(a) and peripheral arterial disease, diagnosis of PFH and CHF, and treatment with iPCSK9 and statins + ezetimibe (Table 2).

## Discussion

Elevated levels of Lp(a) have been associated with a higher risk for vascular disease, early ischemic heart disease, and aortic stenosis [4, 5]. There are no specific treatments currently available for reducing Lp(a) concentrations. However, the clinical characterization of these patients may contribute to vascular risk reclassification. This would favor the optimization of therapeutic measures and targets aimed at reducing the occurrence of atherosclerotic cardiovascular complications [25].

In this study, an evaluation was performed of the clinical characteristics of 878 patients with elevated levels of Lp(a) attended in the vascular risk unit of a tertiary hospital of the Autonomous Community of Madrid. In our study, levels of Lp(a)≥50 mg/dL were associated with a history of dyslipidemia and a lower prevalence of active smoking. In contrast, there were no statistically significant differences in relation to familial history of early cardiovascular disease. This finding contradicts the results of the study by Quyyumi et al. [26], who observed a joint association between familial history of cardiovascular disease along with elevated levels of Lp(a), and long-term cardiovascular risk.

In the present study, elevated levels of Lp(a) were associated with a history of peripheral artery disease. Notably, there were no differences in the prevalence of other previous cardiovascular diseases. This lack of significance may be due to the fact that our patients were attended in a specific vascular risk consultation, which may have resulted in a selection bias that may have influenced results. Indeed, more than half of our patients were on secondary prevention (patients with a previous event: stroke, ischemic heart disease or peripheral artery disease).

In addition, a diagnosis of PFH/CHF was associated with higher levels of Lp(a). Thus, the number of patients with PFH/CHF was four times higher in patients with elevated Lp(a), as compared to patients with normal Lp(a) levels. Although no statistically significant differences were observed in relation to HFH, it could be assumed that the finding of elevated Lp(a) is associated with a diagnosis of familial hyperlipemias, as reported in the literature [27].

The presence of elevated levels of Lp(a) is not associated with a more frequent prescription of high-potency statins. However, a high percentage of the study patients were treated with these drugs in the two groups (>50 %). Of note, the use of a more intensive lipid-lowering therapy was more frequent in patients with elevated Lp(a) (higher combined use of ezetimibe + statins + PCSK9i). This finding suggests that, in compliance with European Society of Cardiology (ESC) guidelines, and due to the high cardiovascular risk of these patients, clinicians aim to reach a lower LCDc target. Hence, the high vascular risk of these patients [28] and the absence of specific therapies available for reducing Lp(a) lead clinicians establish more strict therapeutic targets. The most recent clinical trials demonstrate that PCSK9i are effective in reducing LDL-C and Lp(a) [29, 30], with reported 55 and 25 % reductions, respectively [31, 32]. The clinical benefit of reducing LDL-C and Lp(a) has not yet been demonstrated. However, tendencies seen to suggest its efficacy in reducing major cardiovascular events [33].

Our study has some limitations. The retrospective design of the study limits access to some variables, such as familial history of early cardiovascular disease, with 423 missing data. This may explain our failure to find significant differences in this variable between study groups. Due to the observational nature of the study, relations of causality could not be established, only associative hypotheses. The results obtained do not define specific clinical characteristics that help identify patients with elevated levels of Lp(a) among patients attended due to their high vascular risk. However, the large size and heterogeneity of the sample enabled us to meet the goal of the study.

In conclusion, in a sample of patients attended due to their high vascular risk in a tertiary hospital, elevated levels of Lp(a) were associated with a diagnosis of peripheral arterial disease, PFH and CHF, and with the use of more intensive lipid-lowering treatments.
